# Long-term sick leave for back pain, exposure to physical workload and psychosocial factors at work, and risk of disability and early-age retirement among aged Swedish workers

**DOI:** 10.1007/s00420-022-01862-8

**Published:** 2022-04-22

**Authors:** Angelo d’Errico, Daniel Falkstedt, Melody Almroth, Kathryn Badarin, Tomas Hemmingsson, Katarina Kjellberg

**Affiliations:** 1 Department of Epidemiology, Local Health Unit ASL TO 3, Via Sabaudia 164, 10095 Grugliasco, Turin Italy; 2 Institute of Environmental Medicine, Karolinska Institutet, Stockholm, Sweden; 3 Department of Public Health Sciences, Stockholm University, Stockholm, Sweden; 4 Centre for Occupational and Environmental Medicine, Region Stockholm, Stockholm, Sweden

**Keywords:** Premature retirement, Sickness absence, Back pain, Physical workload, Psychosocial hazards, Work

## Abstract

**Purpose:**

To assess the risk of disability and early-age retirement associated with previous long-term sickness absence for back pain (back-pain SA), exposure to high physical workload, low job control, high demands and high strain, and to evaluate effect modification by work factors on the relationship between back-pain SA and premature retirement.

**Methods:**

All employed Swedish residents born 1946–1955 (*n* = 835,956) were followed up from 2010 to 2016 for disability (DP) and early-age pension (EAP). Associations of premature retirement with exposure to work factors and back-pain SA in the 3 years before follow-up were estimated through proportional hazards models. Retirement, back-pain SA and covariates were assessed through administrative sources, and exposure to work factors through a job-exposure matrix.

**Results:**

In both genders, back-pain SA was associated with DP (> 1 episode: HR 3.23 among men; HR 3.12 among women) and EAP (> 1 episode: HR 1.24 among men; HR 1.18 among women). Higher physical workload and lower job control were also associated with an increased DP risk in both genders, whereas higher job demands showed a decreased risk. For EAP, associations with work factors were weak and inconsistent across genders. No effect modification by work factors was found, except for a negative effect modification by job strain on DP risk among women, i.e. a reduced effect of back-pain SA with increasing exposure.

**Conclusion:**

Back-pain SA was a significant predictor of both DP and EAP, while work factors were consistently associated only with DP. Our results indicate that the joint effect of back-pain SA and work factors on DP is additive and does not support effect modification by work factors.

**Supplementary Information:**

The online version contains supplementary material available at 10.1007/s00420-022-01862-8.

## Introduction

Labor market participation among older Swedish workers has increased in the last decades (Laun and Palme 2019). That said, almost one-quarter of employed 55–64-year-olds left paid work in 2019 (OECD 2019). Early exit from the labour market is more common among women than men (OECD 2019) and blue-collar than white-collar workers (Kadefors et al. 2019).

In most European countries, early retirement and disability retirement are the most common ways of exiting the workforce prematurely (Reeuwijk et al. 2017). In Sweden, disability pension (DP) may be granted to all residents 30–64 years old, if their work ability is reduced by at least 25% due to illness or injury, and it covers up to 65% of an individual’s income loss (Forsakringskassan.se 2020). Early-age pension (EAP) can be taken by any person from 61 years of age. In Sweden, the criteria for granting DPs were tightened in 2006, which led to a decrease in the number of approved DP applications (by approximately 70% from 2006 to 2011) and an increase in EAP (almost three times more frequent from 2004 to 2011 among men and women 55–64 years old) (Kadefors et al. 2019).

Several societal, individual and work-related factors have been associated with early exit from the labour market, including more generous pension legislations, lower income, lower socioeconomic position (SEP), having a retired partner, worse health and higher exposure to physical and psychosocial hazards (Hasselhorn and Apt 2015). In particular, being affected by chronic diseases or functional limitations are important reasons for exiting paid employment (van den Berg et al. 2010; Schuring et al. 2013; Laires et al. 2018).

Musculoskeletal disorders (MSDs) are the most common chronic health conditions among ageing people and MSD incidence increases sharply among those above 50–55 years old (Bot et al. 2005; Thomas et al. 2014; Palmer and Goodson, 2015). The back and upper limbs are the most commonly affected anatomical sites (Punnett and Wegman 2004). The reported prevalence of chronic or disabling back pain in the older European population (10–15%) is around 10–15% (Gourmelen et al. 2007; Gouveia et al. 2016; Parsons et al. 2007). According to 2019 data from the Global Burden of Disease Study, back pain is the leading cause for years lived with disability worldwide in both men and women (https://www.thelancet.com/lancet/visualisations/gbd-compare. Accessed on June 27, 2021). The main risk factors for back pain are exposure to biomechanical loads at work, with an estimated population attributable proportion of approximately 25% (Driscoll et al. 2014), obesity, physical inactivity, smoking, female gender, depressive symptoms, and genetic factors (Maher et al. 2017; Knezevic et al. 2021). The most common disorders causing back pain are intervertebral disc degeneration, with or without radicular pain from nerve root compression, followed by less common pathologies, such as spinal stenosis, facet arthropathy, and spondyloarthropathies (Knezevic et al. 2021). However, no underlying cause is found in the great majority of cases, and large inconsistencies have been reported between radiographic alterations and presence or severity of symptoms (Khan et al. 2017).

A meta-analysis on health as a determinant of early exit from work found that MSDs more than doubled the risk of DP and increased EAP by 20%, though the latter was not significant (van Rijn et al. 2014). Specifically, back pain has been consistently associated with an increased risk of exit through DP (Krause et al. 1997; Borg et al. 2004; Jensen et al. 2012; Dorner et al. 2015; Rahman et al. 2019). However, back pain was not associated with EAP in the few available studies (Jensen et al. 2012; Lallukka et al. 2018; Rice et al. 2011).

Work-related hazards have also been associated with early exit from the labour market. Heavy physical workload has been associated with DP (Krause et al. 1997; Krokstad et al. 2002; Friis et al. 2008; Lahelma et al. 2012; Kjellberg et al. 2016; Falkstedt et al. 2021) and EAP (Lund et al. 2001; Lund and Villadsen 2005; Friis et al. 2007; Sundstrup et al. 2018). A review of 39 studies found moderate evidence of an association between low job control or job strain and DP, but limited evidence for other psychosocial factors (e.g., job demands, effort–reward imbalance, low social support, and repetitive work) (Knardahl et al. 2017). Non-disability retirement has also been associated with low job control or its sub-dimensions (Lund and Villadsen 2005; Friis et al. 2007; Robroek et al. 2013; Thorsen et al. 2016). Furthermore, a recent review showed associations between low job control or low social support and EAP (Browne et al. 2018).

To our knowledge, no studies have investigated the modifying effect of exposure to physical or psychosocial hazards on the relationship between back pain and DP or EAP. At least for several known work-related ergonomic exposures at work which are known risk factors for the development of back pain (e.g. bending and twisting the body, heavy lifting and awkward postures (da Costa and Vieira 2010), we expect, for analogy, that back pain would be aggravated by a continuation of exposure to these factors. Such an increase in symptoms severity would lead to a decreased work ability and greater difficulties in performing physical work duties, in a vicious circle, which, in turn, would increase the likelihood of disability and early retirement. This theoretical model appears supported by the results of different studies showing interactive effects of exposure to high physical workload and back or musculoskeletal pain on reduced work ability or sickness absence (Sundstrup and Andersen 2017; Nygaard et al. 2020).

However, the model proposed may also be valid for psychosocial factors. A German study reported a positive interaction between back pain and stress perception on declining work ability (Oberlinner et al. 2015). Also, in a sample of Chinese workers with back symptoms in the previous year, an increased risk of SA due to back pain was found among workers exposed to high job demand and high effort (Yu et al. 2015).

The aim of this study is two-fold. First, to estimate the separate associations of sickness absence for back pain (back-pain SA), heavy physical workload or psychosocial work factors with an exit from paid employment through premature retirement (DP or EAP). Second, to evaluate effect modification of exposure to physical workload and psychosocial factors at work on the relationship between back-pain SA and premature retirement. Based on previous research, we hypothesize that: (1) both back-pain SA and exposure to heavy physical workload and psychosocial factors will increase the risk of DP and EAP, and: (2) exposure to high physical workload and to low job control, high demand and high strain will interact synergistically with back-pain SA to increase the risk DP and EAP above their additive effect.

## Materials and methods

### Data collection

#### Study population

**Fig. 1:**
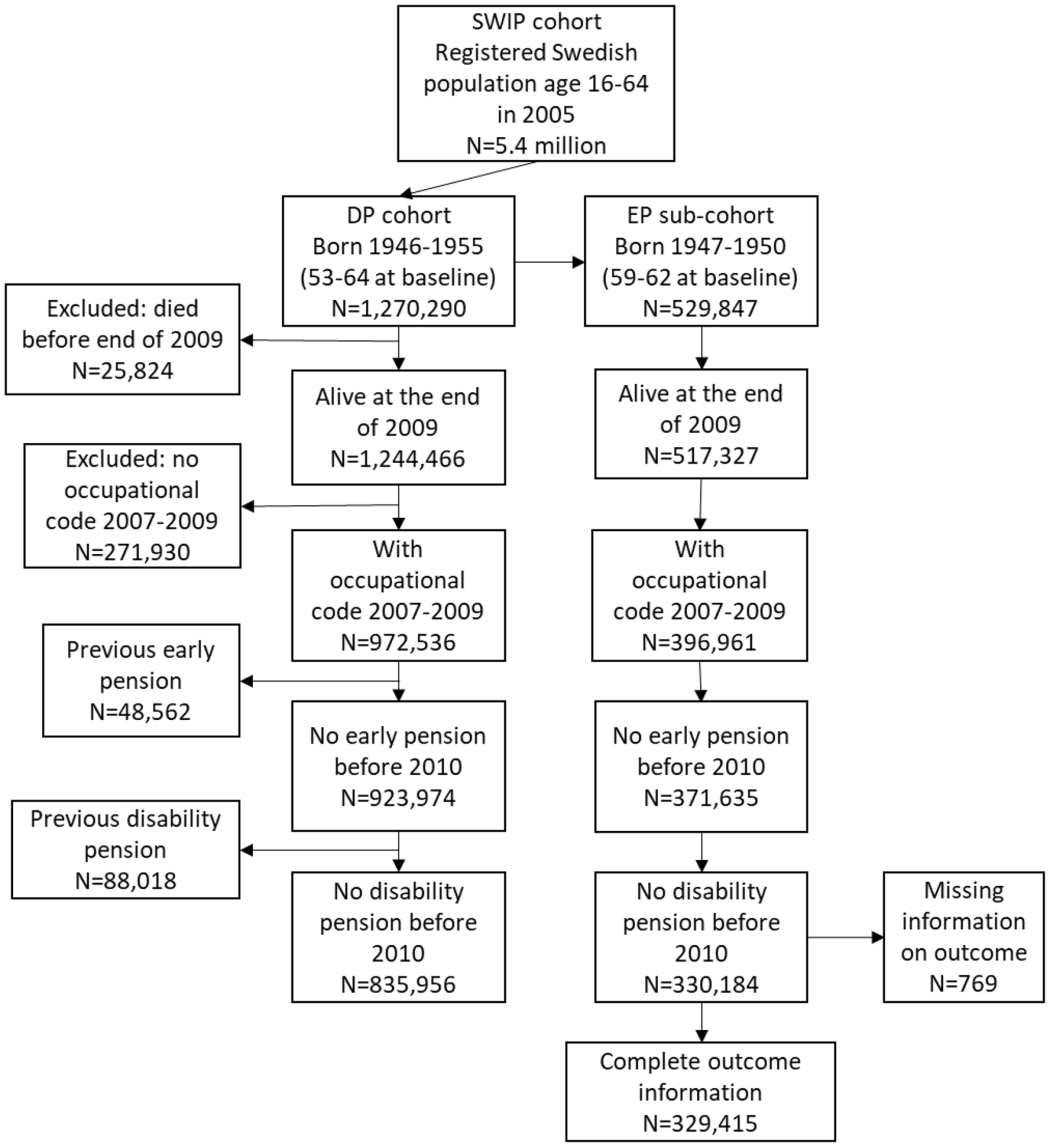
Flow chart on selection of subjects in the cohort and the sub-cohort

This study used data from the Swedish Work, Illness and Labour Market Participation (SWIP) cohort (Falkstedt et al. 2021). The SWIP cohort consists of linked national registers and includes all persons aged 16–64 registered in Sweden in 2005, i.e. around 5.4 million subjects. The sample included all subjects alive at the end of 2009 and employed during 2007–2009; the inclusion criterion of continuous employment was limited to 3 years before follow-up to reduce the potential health-related selection of workers out of employment. Analysis exploring DP (main cohort) included subjects born during 1946–1955 (53–64 years in 2010, the start of follow-up). The analysis exploring early retirement (EAP) (sub-cohort) included subjects born during 1947–1950 (59–62 years at the start of follow-up). The latter restriction was made to capture workers eligible for EAP during the follow-up, i.e. from age 61. The main cohort and the sub-cohort were followed from the beginning of 2010 to the end of 2016. After excluding subjects with DP or EAP before the start of follow-up and those with missing information on occupation (main cohort: *n* = 343,857; sub-cohort: *n* = 156,958), the main cohort included 835,956 subjects and the sub-cohort 328,941 subjects (Fig. 1). Person-time was counted from the start of 2010 until the outcome of interest, death, migration from Sweden, turning 65 years old, or the end of follow-up (end of 2016), whichever came first.

#### Registers and variables

Data for this study were sourced from two registers. The Longitudinal Integration Database for Health Insurance and Labour Market Studies (LISA) register contains information from 1990 onwards for all subjects 16–64 years old on age, sex, education, family conditions, occupation, income, and pensions. Occupations are classified at the four-digit level according to the Swedish classification of occupations (SSYK96), based on the ISCO-88. The Social Insurance Micro Data for Analysis of the Social Insurance System (MIDAS) register contains information on welfare benefits, such as sick leave and DP.

#### Exposures

##### Back pain

Data on sickness absence associated with back pain (back-pain SA) were taken from the MIDAS-register, which provides information on episodes of SA longer than 14 days. In Sweden, SA benefits are paid by the employer from the second to the 14th day of SA, and, afterwards, by the Social Insurance Agency, with a medical certificate required to attest the disease after the first seven days.

The presence of back pain was defined as any episode of long-term SA in the 3 years before the start of follow-up (2007–2009) for “Thoracic, thoracolumbar, and lumbosacral intervertebral disc disorders” (ICD-10 code: M51) or “Dorsalgia” (ICD-10 code: M54). Sickness episodes for the disease codes M51 and M54 were selected, as these disease groups include the disorders that are main causes of back pain: intervertebral disc degeneration (Risbud and Shapiro 2014), with or without nerve root compression (Van Boxem et al. 2010), and non-specific low back pain (Maher et al. 2017). The detail of the available codes of disease was only at 2 digits, so it was not possible to distinguish between thoracic, thoracolumbar, and lumbosacral sites, although the lumbar region is by far the site most frequently affected by pain (Singh et al. 2012).

##### Heavy physical workload

Exposure to heavy physical workload was estimated through a Swedish job-exposure matrix (JEM). The JEM was constructed on data from the Swedish Work Environment Surveys 1997–2013, and its development has been described previously (Badarin et al. 2021). The JEM provides information on gender-specific mean scores on the proportion of work time exposed to eight work-related physical hazards for 355 occupations.

This study investigates five work-related physical hazards that potentially increase the risk of back pain—heavy lifting (≥ 15 kg), forward bent position, twisted trunk posture, frequent bending or twisting of the trunk, and physically strenuous work. A strong correlation was found between these physical hazards (e.g., Spearman rho = 0.71 for the correlation between frequent bending and twisting of the trunk and heavy lifting to rho = 0.94 for the correlation between heavy lifting and overall physically strenuous work), precluding the possibility to meaningfully assess the independent association of each factor with premature retirement. Therefore, a composite index of physical workload was created by combining the average mean scores of the different items (Cronbach alpha: men = 0.97, women = 0.96).

##### Work-related psychosocial hazards

Exposure to three work-related psychosocial hazards was assigned to the study population using a Swedish psychosocial JEM. The JEM was created on the same data source as the physical JEM. Its construction and the details of the individual psychosocial hazards have been described previously (Almroth et al. 2021). The psychosocial factors explored in this study include job demands, job control and job strain, the latter computed as demand scores divided by control scores, a method used in previous studies (Choi et al. 2008; d’Errico et al. 2011).

A mean score for each aforementioned physical and psychosocial exposure was assigned to all participants of this study based on occupations (4-digit SSYK96 job codes) held during the 3 years (2007–2009) before the start of follow-up. Estimated exposure scores for each year were averaged across the 3 years to construct mean levels of exposure to each physical and psychosocial hazard.

#### Outcomes

Premature retirements through DP and EAP were investigated separately. Information on type and timing of each outcome was drawn from the MIDAS and the LISA registers, respectively. In this study, DP was defined as a first episode (full or partial) of DP due to any health-related condition. Subjects who took an EAP were identified as those who began receiving an age-related pension from the age of 61 (before turning 65) and earned an income from work smaller than one Price Basic Amount (PBA) for the following year. The yearly PBA ranged from 4,194 Euros to 4,382 Euros during the follow-up period, information found at Statistics Sweden’s website (scb.se 2021).

#### Covariates

Information on age, gender, marital status, and education was obtained from the LISA register during the first baseline year (2007) as well as previous unemployment during 2005–2009. Age was categorized into three groups by birth year for the main cohort (1946–1948, 1949–1951, and 1952–1955) and two groups for the sub-cohort (1947–1948, and 1949–1950). Four groups indicated marital status (married, unmarried, divorced, and widowed). Educational attainment was grouped according to years of education (≤ 9, 10–11, 12, 13–14, and ≥ 15). Previous unemployment was categorized into three groups (no unemployment days, unemployment ≤ 365 days, unemployment > 365 days).

### Data analysis

The separate associations of premature retirement (DP and EAP) with back pain and work-related hazards were estimated through Cox proportional hazards models.

The number of back-pain SA episodes (lasting longer than 14 days) during 2007–2009 was categorized into three groups (i. none, ii. one episode, iii. ≥ two episodes). To investigate associations between exposure to work-related hazards (composite physical workload index, job demands, job control and job strain) and the outcomes, the mean JEM scores were divided into tertiles (low, medium and high).

All analyses were stratified by gender. Our first model (Model 1) only adjusted for age, while in the second step (Model 2), marital status, previous unemployment, and highest achieved education were added. Because work factors were quite strongly correlated with each other and educational level, no mutual adjustment for work factors was made to avoid distortions of the hazard ratios due to multicollinearity. In fact, according to Vatcheva et al. (2016), the inclusion of several independent variables strongly correlated with each other in a single model would lead to substantial multicollinearity, which would not be identified through standard methods (e.g., Variance Inflation Factor). Of note, among men, the correlation between educational level and heavy physical workload was as high as Spearman’s rho = − 0.53. Socio-economic position, or its proxy indicator educational attainment, has been acknowledged as an independent predictor of premature retirement (Schuring et al. 2013; Venti and Wise 2015), which precluded the possibility of excluding education from the adjustment variables.

Trends in risk of DP and EAP across increasing categories of back-pain SA and exposure to work factors were also examined through Wald tests. All associations showed significant linear trends except for the job strain variable according to disability pension among men.

The potential modifying effect of exposure to physical workload and psychosocial factors on the association between back pain and the two outcomes was assessed by cross-tabulating HRs of DP and EAP by categories of back-pain SA (0, 1, 2 + episodes) and work factors (tertiles) from fully adjusted multivariate Cox models, testing the interaction between each workplace factor and back-pain SA by means of a Wald test for each interaction term.

In a sensitivity analysis, we also examined associations of back-pain SA with DP and EAP, as well as effect modification by work factors, using categories of back-pain SA in days of absence (no back-pain SA, ≤ 90 days, > 90 days), to assess whether days of back-pain SA were better predictors of premature retirement than number of back-pain SA episodes.

Because there was statistical evidence of non-proportional hazards, we stratified the analysis by 2 years increments of follow-up time. While there was some variation in the estimates, there was no clear pattern of divergence from the main analysis over the 2 years increments. Hazard ratios are thus interpreted as the weighted average ratio over the follow-up period.

## Results

### Descriptive

During follow-up, 11,172 men (2.6%) and 14,166 women (3.5%) were granted DP (main cohort) (Table 1, left side). In both genders, the highest prevalence of DP was found among subjects with lower education or previous unemployment. Subjects who received DP showed a prevalence of back-pain SA three times higher than those who did not.

**Table 1:** Distribution of baseline covariates according to disability pension and early old age pension for men and women

	Main cohort				Sub-cohort			
	Men		Women		Men		Women	
	DP *N* (%)	No DP *N* (%)	DP *N* (%)	No DP *N* (%)	Pension *N* (%)	No pension *N* (%)	Pension *N* (%)	No pension *N* (%)
Birth year								
1946–1948	1238 (11)	111,034 (27)	1301 (9)	103,760 (26)	11,731 (39)	67,190 (48)	11,944 (40)	62,333 (48)
1949–1951	3604 (32)	129,888 (31)	4375 (31)	124,431 (31)	18,241 (61)	71,491 (52)	17,707 (60)	68,778(52)
1952–1955	6330 (57)	173,951 (42)	8490 (60)	167,554 (42)	–	–	–	–
Education								
> 15	1160 (10)	78,906 (19)	2313 (16)	88,685 (22)	4023 (13)	28,670 (21)	4781 (16)	30,923 (24)
13–15	1203 (11)	57,237 (14)	2217 (16)	67,064 (17)	3846 (13)	17,678 (13)	4322 (15)	21,038 (16)
12	1506 (14)	64,032 (15)	1444 (10)	42,108 (11)	5535 (18)	23,456 (17)	3072 (10)	12,711 (10)
10–11	4042 (36)	119,050 (29)	5655 (40)	140,521 (36)	8640 (29)	35,785 (26)	11,634 (39)	46,028 (35)
≤ 9	3235 (29)	94,986 (23)	2513 (18)	57,049 (14)	7902 (26)	32,848 (24)	5831 (20)	20,291 (15)
Civil status								
Married	5839 (52)	260,339 (63)	7687 (54)	244,869 (62)	19,276 (64)	90,828 (65)	21,695 (73)	79,721 (61)
Unmarried	2867 (26)	81,042 (20)	2381 (17)	58,994 (15)	5347 (18)	22,988 (17)	3020 (10)	17,231 (13)
Divorced	2314 (21)	68,565 (17)	3,634 (26)	78,140 (20)	4861 (16)	22,864 (16)	4230 (14)	28,032 (21)
Widowed	152 (1)	4927 (1)	464 (3)	13,742 (3)	488 (2)	2,001 (1)	706 (2)	6127 (5)
Unemployment days								
None	7842 (70)	350,845 (85)	10,724 (76)	344,451 (87)	23,300 (78)	119,763 (86)	24,299 (82)	115,992 (89)
≤ 365	2160 (19)	42,558 (10)	2411 (17)	36,025 (9)	4089 (14)	12,358 (9)	3491 (12)	10,430 (8)
> 365	1162 (10)	21,098 (5)	1027 (7)	15,020 (4)	2556 (9)	6447 (5)	1841 (6)	4609 (4)
Sickness episodes								
None	10,198 (91)	402,662 (97)	13,019 (92)	382,966 (97)	28,879 (96)	134,536 (97)	28,466 (96)	126,905 (97)
(M51 & M54)								
1	772 (7)	10,210 (2)	890 (6)	10,729 (3)	890 (3)	3474 (3)	1002 (3)	3553 (3)
≥ 2	202 (2)	2001 (0)	257 (2)	2050 (1)	203 (1)	671 (0)	183 (1)	653 (1)
Physical workload								
Low	2203 (20)	144,491 (35)	3378 (24)	140,854 (36)	9907 (33)	50,109 (36)	9475 (32)	49,407 (38)
Med	3854 (35)	138,258 (33)	4804 (34)	137,275 (35)	10,478 (35)	45,270 (33)	10,566 (36)	44,163 (34)
High	5115 (46)	132,124 (32)	5984 (42)	117,616 (30)	9587 (32)	43,302 (31)	9610 (32)	37,541 (29)
Job strain								
Low	3947 (35)	136,269 (33)	4322 (31)	132,086 (33)	9675 (32)	45,895 (33)	10,191 (34)	44,806 (34)
Medium	3304 (30)	140,003 (34)	4842 (34)	130,504 (33)	10,846 (36)	46,689 (34)	10,024 (34)	42,975 (33)
High	3921 (35)	138,601 (33)	5002 (35)	133,155 (34)	9451 (32)	46,097 (33)	9436 (32)	43,330 (33)
Control								
Low	4627 (41)	135,463 (33)	5523 (39)	130,599 (33)	9091 (30)	44,964 (32)	9997 (34)	42,533 (32)
Medium	4169 (37)	137,329 (33)	5294 (37)	129,605 (33)	10,370 (35)	45,433 (33)	10,090 (34)	42,332 (32)
High	2376 (21)	142,081 (34)	3349 (24)	135,541 (34)	10,511 (35)	48,284 (35)	9564 (32)	46,246 (35)
Demands								
Low	5120 (46)	132,058 (32)	5591 (39)	120,278 (30)	9973 (33)	43,318 (31)	10,665 (36)	40,091 (31)
Medium	3243 (29)	140,541 (34)	4737 (33)	135,137 (34)	9833 (33)	47,160 (34)	10,172 (34)	44,121 (34)
High	2809 (25)	142,274 (34)	3838 (27)	140,330 (35)	10166 (34)	48,203 (35)	8814 (30)	46,899 (36)

*DP* disability pension,* M51* other intervertebral disc disorders,* M54* dorsalgia

In the sub-cohort, 29,972 men (17.8%) and 29,651 women (18.4%) received an EAP during follow-up (Table 1, right side). As for DPs, both men and women who accessed EAP were less educated, compared to those who remained at work, whereas the prevalence of back-pain SA was similar in the two groups.

### Back pain, work factors, and risk of disability pension

In both genders, the analyses adjusted only for age showed strong and significantly increased risks of DP associated with back-pain SA, higher physical workload, lower job control, and lower job demands (Table 2, Model 1). Among women, a slightly increased risk of DP was associated with job strain, whereas among men medium job strain showed a reduced risk.

**Table 2:** Hazard ratios and 95% confidence intervals for disability pension according to back pain sickness episodes (back-pain SA) and physical and psychosocial workplace factors

	*N* cases (%)	Model 1	Model 2
*Men*			
Back-pain SA			
None	10,198 (2)	1	1
1	772 (7)	2.89 (2.69–3.11)	2.47 (2.30–2.66)
≥ 2	202 (9)	3.80 (3.31–4.37)	3.23 (2.81–3.71)
Physical workload			
Low	2203 (2)	1	1
Med	3854 (3)	1.79 (1.70–1.89)	1.44 (1.36–1.52)
High	5115 (4)	2.46 (2.34–2.59)	1.75 (1.65–1.85)
Job strain			
Low	3947 (3)	1	1
Med	3304 (2)	0.82 (0.79–0.86)	0.89 (0.85–0.93)
High	3921 (3)	0.97 (0.93–1.02)	1.02 (0.97–1.06)
Job control			
Low	4627 (3)	1.99 (1.89–2.09)	1.58 (1.50–1.66)
Med	4169 (3)	1.79 (1.67–1.88)	1.43 (1.36–1.51)
High	2376 (2)	1	1
Job demands			
Low	5120 (4)	1	1
Med	3243 (2)	0.60 (0.58–0.63)	0.75 (0.72–0.78)
High	2806 (2)	0.52 (0.50–0.54)	0.70 (0.67–0.74)
*Women*			
Back-pain SA			
None	13,019 (3)	1	1
1	890 (8)	2.38 (2.23–2.55)	2.21(2.06–2.36)
≥ 2	257 (11)	3.42 (3.03–3.87)	3.12 (2.76–3.53)
Physical workload			
Low	3378 (2)	1	1
Med	4804 (3)	1.42 (1.36–1.48)	1.34 (1.28–1.40)
High	5984 (5)	2.05 (1.97–2.14)	1.75 (1.67–1.84)
Job strain			
Low	4322 (3)	1	1
Med	4842 (4)	1.11 (1.07–1.16)	1.12 (1.08–1.17)
High	5002 (4)	1.11 (1.07–1.16)	1.26 (1.21–1.32)
Job control			
Low	5523 (4)	1.67 (1.60–1.74)	1.58 (1.51–1.65)
Med	5294 (4)	1.61 (1.54–1.68)	1.49 (1.42–1.56)
High	3349 (2)	1	1
Job demands			
Low	5591 (4)	1	1
Med	4737 (3)	0.73 (0.71–0.76)	0.81 (0.78–0.85)
High	3838 (3)	0.57 (0.55–0.60)	0.74 (0.70–0.78)

*Back-pain SA *n. episodes of sickness absence for back pain

Model 1 is adjusted for age

Model 2 is adjusted for age, civil status, previous unemployment, and highest achieved education

In the analyses adjusted for the other covariates (Table 2, Model 2), the associations found in Model 1 remained statistically significant in both genders, except for job strain, with greater risk attenuations among men.

#### Effect modification by work factors of the association between back-pain SA and DP risk

Cross-tabulation of HRs by categories of back-pain SA and exposure to work factors showed in general additive effects of work exposures and back-pain SA on the risk of DP (Table 3). No significant effect modification by work factors was found among men, while among women, a reduced effect of back-pain SA with increasing exposure to job strain was observed (*p* = 0.001).

**Table 3:** Hazard ratios and 95% confidence intervals for disability pension according to combinations of back pain sickness episodes (back-pain SA) and physical and psychosocial workplace factors

Back-pain SA	None	1	≥2	*p* value
*Men*				
Physical workload				
Low	1	2.24 (1.72–2.91)	5.04 (3.17–8.01)	0.1042
Med	1.40 (1.32–1.49)	3.62 (3.17–4.14)	4.23 (3.28–5.47)	
High	1.68 (1.59–1.79)	3.81 (3.43–4.24)	4.85 (4.03–5.83)	
Job strain				
Low	1	2.31 (2.04–2.61)	2.93 (2.33–3.69)	0.3218
Med	0.93 (0.88–0.98)	2.10 (1.82–2.43)	3.43 (2.65–4.43)	
High	1.01 (0.97–1.06)	2.51 (2.22–2.83)	2.76 (2.17–3.50)	
Job control				
Low	1.55 (1.47–1.64)	3.55 (3.18–3.96)	4.51 (3.69–5.52)	0.1167
Med	1.42 (1.35–1.50)	3.38 (2.98–3.82)	4.32 (3.42–5.43)	
High	1	2.88 (2.36–3.51)	4.33 (2.96–6.33)	
Job demands				
Low	1	2.22 (2.01–2.45)	3.03 (2.53–3.63)	0.1452
Med	0.75 (0.72–0.79)	1.94 (1.68–2.23)	2.46 (1.87–3.22)	
High	0.71 (0.68–0.75)	2.00 (1.69–2.37)	2.29 (1.58–3.31)	
*Women*				
Physical workload				
Low	1	2.47 (2.06–2.96)	2.27 (1.43–3.60)	0.1888
Med	1.32 (1.26–1.39)	2.78 (2.44–3.16)	4.12 (3.27–5.20)	
High	1.70 (1.62–1.78)	3.36 (3.05–3.69)	4.85 (4.14–5.68)	
Job strain				
Low	1	2.52 (2.23–2.85)	3.14 (2.47–4.00)	0.0011
Med	1.04 (0.99–1.08)	2.07 (1.84–2.32)	2.84 (2.30–3.51)	
High	1.04 (0.99–1.09)	1.92 (1.71–2.16)	2.95 (2.41–3.59)	
Job control				
Low	1.55 (1.48–1.62)	2.98 (2.69–3.31)	4.53 (3.82–5.38)	0.1049
Med	1.47 (1.40–1.54)	3.22 (2.89–3.60)	4.19 (3.40–5.16)	
High	1	2.50 (2.10–2.97)	3.25 (2.27–4.66)	
Job demands				
Low	1	2.11 (1.90–2.33)	2.84 (2.37–3.40)	0.4345
Med	0.81 (0.78–0.84)	1.90 (1.70–2.12)	2.66 (2.17–3.26)	
High	0.75 (0.71–0.79)	1.55 (1.31–1.84)	2.47 (1.81–3.36)	

*Back-pain SA *n. episodes of sickness absence for back pain

Models adjusted for age, civil status, previous unemployment, and education

*p *value corresponds to Wald test for interaction term

### Back pain, work factors, and risk of Early Age Pension

In the age-adjusted analysis, EAP was associated with back-pain SA and physical workload, although the hazard ratios were much lower than those observed for DP (Table 4, Model 1).

**Table 4:** Hazard ratios and 95% confidence intervals for early old age pension without income (1 PBA) according to back pain sickness episodes (back-pain SA) and physical and psychosocial workplace factors

	*N* cases (%)	Model 1	Model 2
*Men*			
Back-pain SA			
None	28,879 (18)	1	1
1	890 (20)	1.16 (1.08–1.24)	1.07 (1.00–1.14)
≥2	203 (23)	1.36 (1.19–1.56)	1.24 (1.08–1.42)
Physical workload			
Low	9907 (17)	1	1
Med	10,478 (19)	1.15 (1.12–1.18)	0.94 (0.91–0.96)
High	9587 (18)	1.10 (1.07–1.13)	0.82 (0.80–0.85)
Job strain			
Low	9675 (17)	1	1
Med	10,846 (19)	1.09 (1.06–1.12)	1.13 (1.10–1.17)
High	9451 (17)	0.98 (0.95–1.00)	1.02 (0.99–1.05)
Job control			
Low	9091 (17)	0.94 (0.91–0.96)	0.83 (0.81–0.86)
Med	10,370 (19)	1.04 (1.01–1.07)	0.91 (0.89–0.94)
High	10,511 (18)	1	1
Job demands			
Low	9973 (19)	1	1
Med	9833 (17)	0.92 (0.89–0.94)	1.05 (1.02–1.08)
High	10,166 (17)	0.93 (0.90–0.96)	1.14 (1.11–1.17)
*Women*			
Back-pain SA			
None	28,466 (18)	1	1
1	1002 (22)	1.22 (1.15–1.30)	1.16 (1.09–1.24)
≥2	183 (22)	1.23 (1.06–1.42)	1.18 (1.02–1.36)
Physical workload			
Low	9475 (16)	1	1
Med	10,566 (19)	1.22 (1.18–1.25)	1.11 (1.07–1.14)
High	9610 (20)	1.29 (1.25–1.33)	1.07 (1.03–1.10)
Job strain			
Low	10,191 (19)	1	1
Med	10,024 (19)	1.02 (0.99–1.05)	1.03 (1.01–1.06)
High	9436 (18)	0.95 (0.93–0.98)	1.07 (1.04–1.10)
Job control			
Low	9997 (19)	1.11 (1.08–1.15)	1.09 (1.06–1.12)
Med	10,090 (19)	1.13 (1.10–1.16)	1.07 (1.04–1.10)
High	9564 (17)	1	1
Job demands			
Low	10,665 (21)	1	1
Med	10,172 (19)	0.87 (0.85–0.90)	0.94 (0.91–0.96)
High	8814 (16)	0.73 (0.71–0.75)	0.96 (0.93–1.00)

*PBA *price basic amount*, Back-pain SA *n. episodes of sickness absence for back pain

Model 1 is adjusted for age

Model 2 is adjusted for age, civil status, previous unemployment, and highest achieved education

Exposure to high job demands was inversely associated with EAP. Exposure to medium job control increased the risk of EAP in both genders, but associations between low job control and EAP went in opposite directions for men and women. High job strain was associated with a slightly decreased risk in both genders, while medium job strain with an increased risk in men (HR 1.09).

In multivariate models (Table 4, Model 2), the associations between back-pain SA and EAP persisted, although attenuated in strength, with a significant dose–response in both genders. Regarding work factors, among women the associations with physical workload, job control and job demands also were attenuated, but remained statistically significant and in the same direction as in Model 1, whereas among men the associations with high physical workload, job demands and medium job control changed direction.

#### Effect modification by work factors of the association between back-pain SA and EAP risk

In general, associations between back-pain SA and EAP were similar in strength across increasing levels of exposure to work factors. Only a marginally significant negative interaction was observed between back-pain SA and physical workload (*p* = 0.07) among men, i.e. a reduced effect of back-pain SA with increasing levels of physical workload, whereas no effect modification was found among women (Table 5).

**Table 5:** Hazard ratios and 95% confidence intervals for early old age pension without income (1 PBA) according to *combinations of* back pain sickness episodes (back-pain SA) and physical and psychosocial workplace factors

Back-pain SA	None	1	≥2	*p* value
*Men*				
Physical workload				
Low	1	1.14 (0.97–1.35)	1.99 (1.41–2.82)	0.0713
Med	0.93 (0.91–0.96)	1.05 (0.94–1.18)	1.03 (0.80–1.32)	
High	0.82 (0.79–0.85)	0.87 (0.79–0.96)	1.03 (0.86–1.25)	
Job strain				
Low	1	1.05 (0.94–1.17)	1.15 (0.90–1.46)	0.7712
Med	1.13 (1.10–1.17)	1.20 (1.06–1.35)	1.52 (1.20–1.94)	
High	1.02 (0.99–1.05)	1.15 (1.02–1.295)	1.28 (1.01–1.62)	
Job control				
Low	0.83 (0.80–0.85)	0.96 (0.87–1.07)	1.05 (0.85–1.29)	0.2589
Med	0.91 (0.89–0.94)	0.91 (0.81–1.01)	1.22 (0.98–1.50)	
High	1	1.13 (0.98–1.31)	1.11 (0.76–1.62)	
Job demands				
Low	1	1.12 (1.02–1.23)	1.13 (0.92–1.39)	0.4139
Med	1.05 (1.02–1.08)	1.11 (0.98–1.26)	1.55 (1.23–1.96)	
High	1.14 (1.11–1.18)	1.19 (1.02–1.38)	1.43 (1.03–1.98)	
*Women*				
Physical workload				
Low	1	1.23 (1.06–1.43)	1.08 (0.71–1.63)	0.4441
Med	1.09 (1.06–1.13)	1.15 (1.03–1.30)	1.33 (1.03–1.72)	
High	1.04 (1.00–1.08)	1.23 (1.13–1.35)	1.21 (1.00–1.47)	
Job strain				
Low	1	1.17 (1.04–1.33)	1.01 (0.75–1.36)	0.7188
Med	1.03 (1.00–1.06)	1.19 (1.07–1.33)	1.35 (1.07–1.70)	
High	1.06 (1.03–1.10)	1.21 (1.09–1.34)	1.23 (0.97–1.57)	
Job control				
Low	1.08 (1.05–1.12)	1.25 (1.14–1.37)	1.36 (1.11–1.66)	0.8023
Med	1.06 (1.03–1.10)	1.19 (1.07–1.32)	1.17 (0.92–1.50)	
High	1	1.19 (1.03–1.38)	1.02 (0.68–1.52)	
Job demands				
Low	1	1.19 (1.08–1.31)	1.16 (0.94–1.43)	0.9669
Med	0.94 (0.91–0.97)	1.08 (0.97–1.19)	1.13 (0.88–1.44)	
High	0.97 (0.93–1.00)	1.08 (0.94–1.25)	1.12 (0.78–1.62)	

*PBA *price basic amount*, Back-pain SA *n. episodes of sickness absence for back pain

Models adjusted for age, civil status, previous unemployment, and education

*p *value corresponds to Wald test for interaction term

### Sensitivity analysis

Using categories based on the number of days of back-pain SA, in the highest category, the risk of DP increased, while in the intermediate category the risk decreased, compared with the results of the main analysis (Table S1). For EAP, in contrast, using days of back-pain SA instead of the number of spells changed the associations very little in women, while in men, the HR for the highest back-pain SA category decreased, becoming insignificant (Table S2).

No significant effect modification by work factors was found for DP, except for job strain among women, as in the main analysis (Table S3). For EAP, the marginally significant negative interaction observed between the number of back-pain SA spells and physical workload became significant using categories of back-pain SA days (*p* = 0.035) (Table S4).

## Discussion

### Main findings

In this study, back-pain SA was found to be a significant predictor of both DP and EAP among men and women. Dose–response relationships were found between back-pain SA and both outcomes, although stronger associations were found for DP.

Higher physical workload and lower job control were also positively associated with DP among both genders, whereas higher job demands were associated with a decreased risk. Small and inconsistent associations were found for job strain. This could be explained by the opposite effects found for low control and high demands on DP risk, which canceled each other out in the job strain index.

Regarding EAP, in multivariate models, higher physical workload was associated with an increased risk among women only. Job control and job demands showed weak and inconsistent associations with EAP among both men and women.

In contrast with our hypothesis, exposure to physical and psychosocial factors was not found to act synergistically with back-pain SA to increase the risk of DP or EAP. For DP, only a negative effect modification by job strain was observed among women. For EAP, in spite of the modest associations with work factors, a marginally significant negative effect modification by physical workload was found among men, which was significant in the sensitivity analysis based on days of back-pain SA.

### Interpretation of the results

Based on our results, preventive efforts to reduce DP among older workers should concentrate on reducing exposure to high physical workload and low job control.

Exposure to both factors typically stems from the type of work organization and technology characterizing each job; therefore, physical and psychosocial hazards cluster in occupations (McDonald et al. 2001; Kausto et al. 2011). It was therefore not unexpected that, in this study, these factors were strongly correlated, as explained in the methods.

The inverse association between psychological demands and DP risk may be attributable to the fact that many jobs exposed to high quantitative demands are also characterized by higher variety, creativity and workers’ engagement. These job characteristics could decrease the risk of premature exit through DP. Another possible explanation is residual confounding by socioeconomic position. Workers in higher occupational classes are generally exposed to higher job demands and have a lower tendency to apply for DP. Adjustment for educational level may not have eliminated confounding by occupational class.

The higher risk of DP for the category of > 90 days of back-pain SA found in the sensitivity analysis, compared to that observed for the category of ≥ 2 episodes back-pain SA, would indicate that length of back-pain SA is a better predictor of DP than number of episodes.

The cross-tabulation of results for DP by categories of back-pain SA and work factors indicates that the joint effect of work exposures and back-pain SA is mostly additive. However, the negative interaction with job strain among women and the decrease in the risk gradient by increasing exposure to physical workload and low job control among men with more back-pain SA episodes (even though these interactions did not reach statistical significance) suggest the presence of a small negative effect modification exerted by exposure to these factors, especially among men. A possible explanation is that some workers with a greater number of episodes or days of back-pain SA, who likely suffer from more disabling back pain, changed to jobs with lower physical workload or higher job control. It seems likely that when back pain becomes more severe, it tends to disable workers irrespective of their working conditions. Therefore, interventions aimed at reducing exposure of subjects affected by persistent back pain should be implemented in the early phase of the disorder before it becomes chronic and disabling. Back pain derives mainly from the development of an inflammatory process affecting the intervertebral discs or other joints of the spine (Risbud and Shapiro 2014). Biomechanical load of the anatomic structures affected by inflammation is expected to sustain this inflammatory process, increasing the likelihood of development of chronic pain and functional disability (Nieminen et al. 2021).

The low and inconsistent associations between work factors and EAP would indicate that they play a minor role in this type of exit. For EAP, other personal and family factors seem more important determinants of the retirement decision, such as financial situation, partner’s employment status, need of caring for children or sick family members (Hasselhorn and Apt 2015). The change among men in the direction of the slight associations of EAP with heavy physical workload, high psychological demand and medium job control in multivariate analysis appears a result of uncertain significance, although it may be due to multicollinearity between work factors and educational level in the regression model, considering the strong correlation observed with physical workload.

Regarding effect modification by work factors, the significant negative interaction observed between back-pain SA days and physical workload among men in the sensitivity analysis would also suggest a decreased influence of exposure to heavy physical demand for EAP when back pain becomes more severe.

### Comparison with other studies

Generally, our results are consistent with the few existing studies exploring associations between back pain and all-cause DP, which show increased risks for DP according to the presence of back pain or back-pain SA (Krause et al. 1997; Borg et al. 2004; Jensen et al. 2012; Dorner et al. 2015; Rahman et al. 2019). These studies report varying strengths of associations, possibly attributable to differences in the populations investigated (e.g., age) and methods used, particularly how back pain was ascertained and adjustment for different confounders. It appears that stronger associations with DP risk were found when using back-pain SA as an indicator of back disorders rather than self-reported back pain or other methods (administrative records, clinical assessment). It is important to note that long-term sick leaves are granted by the social insurance agency based on reduced work ability, and that long periods of sickness absence almost always predate a DP, as decisions on DP are often based on long problematic sick leave periods. Therefore, workers with long-term back-pain SA are stronger candidates for DP than those with back pain only. The higher risks found in the two studies based on back-pain SA (Borg et al. 2004; Dorner et al. 2015), compared to our results, could be explained by their exploration of a younger population; the reference category in these studies (those without back-pain SA) was likely characterized by a lower incidence of DP than in our study. Furthermore, the study by Borg et al. (2004) used a longer duration of back pain-SA (> 90 days) than in our study. Defining back-pain SA as > 90 days might have contributed to Borg et al.’s higher risk estimates. Interestingly, Borg et al.’s findings were roughly comparable to the risk of DP for back-pain SA > 90 days estimated in our sensitivity analysis.

Only a few studies on the relationship between back pain and EAP have been found, none of which identify an increased probability of EAP, in contrast to this study. A Danish study on nurses’ aides found a decreased risk of EAP, with significantly protective hazard ratios for all categories of pain duration (Jensen et al. 2012), while no association was found in two British studies (Rice et al. 2011; Lallukka et al. 2018). Given that in these studies back pain was self-reported, the discrepancy with our results may stem, as for DP, from differences in back pain ascertainment.

Our results on the relationship between exposure to work factors and DP support findings from previous research that generally show an increased risk of DP following exposure to heavy physical workload and low job control. Most studies exploring physical workload estimated significant excess risks of DP (by 40–100%) in adjusted analyses, with moderate to strong risk attenuations after controlling for covariates (Lahelma et al. 2012; Prakash et al. 2017; Kjellberg et al. 2016; Falkstedt et al. 2021). Furthermore, significant dose–response relationships between higher physical workload and DP risk (Krause et al. 1997; Krokstad et al. 2002; Lahelma et al. 2012; Kjellberg et al. 2016; Sundstrup et al. 2018; Falkstedt et al. 2021) are consistent with our results. For job control, our results appear in line with studies indicating that exposure to low job control has a positive and independent effect on the risk of DP (Knardahl et al. 2017). Existing studies on job demands and DP show conflicting results. Two studies found statistically significant increased risks of DP for exposure to high demands (Canivet et al. 2013; Krokstad et al. 2002). Another study reported a marginally statistically significant decreased risk (Thielen et al. 2014). Such inconsistencies make it difficult to interpret the overall association. Also, the use of a JEM to measure exposure to job demands may have affected the comparability of our results with those of other studies, given that exposure assessment through self-reports may be influenced by poor health or low work ability, and these studies were all based on individual self-reported data.

A few available studies have explored EAP and physical or psychosocial factors at work. A study on male waste collectors and municipal workers reported a six-fold increased risk associated with bending of the back (Lund et al. 2001). Another study found slightly increased risks of EAP among aged workers exposed to extreme bending of neck/back, working mainly standing/squatting and low skill discretion (Lund and Villadsen (2005). Similarly, modest associations have been observed with high physical demand and low influence at work among nurses (Friis et al. 2007). HRs above two-fold were estimated for cumulative exposure to hard and very hard physical work during the whole working life among workers 49–63 years from three different Danish cohorts (Sundstrup et al. 2018). The results of the aforementioned studies are only partially consistent with ours but suggest a weak association between high physical workload or low job control and EAP, which is similar to our findings for women.

To our knowledge, no studies have investigated whether adverse physical or psychosocial factors at work are effect modifiers of the association between back pain and all-cause DP or EAP. However, in a Swedish cohort of twins with back or neck pain, high job control and low job demands were found to decrease the risk of work disability (defined as SA for more than 90 days or DP), after controlling for age, sex, education, self-rated health and mental disorders (Mather et al. 2019).

### Strengths

A major strength of this study is the large population investigated, which allowed the evaluation of effect modification by exposure to work factors on the relationship between back-pain SA and premature retirement with great statistical power. Further, the study population included all employed Swedish residents of selected ages, a feature that implies no errors in sampling design. Third, as information on back-pain SA, DP and EAP was derived from administrative sources, differential misclassification of these variables is unlikely. The use of a JEM to assign exposure to physical workload and psychosocial factors also prevented differential misclassification of exposure, but likely introduced a degree of non-differential misclassification, because exposure scores are amalgamated for each occupational group, without variability within occupations.

### Limitations

A limitation of this study is the lack of adjustment for the presence of other illnesses, which may confound the association between back-pain SA and premature retirement, as people affected by back pain have been found at higher risk of carrying other chronic morbidities, such as musculoskeletal pain in other regions (Øverås et al. 2021) and depressive symptoms (Cartensen et al. 2012).

Furthermore, information on behavioral risk factors (e.g., smoking, physical inactivity and overweight) was not available in the administrative datasets used for the study, which may have played a confounding role in the associations of premature retirement with back-pain SA and with work factors. However, as our analyses were adjusted for educational level, confounding by lifestyle risk factors was partially controlled for, given the strong educational gradient in exposure to such factors in Sweden, like in other countries (Falkstedt et al. 2021; Mäki et al. 2014).

Besides not considering within-job variability of exposure, as said above, JEMs do not allow to take into account the individual subjective experience of exposure to work factors, which may be affected by workers’ characteristics, such as gender, anthropometric measures, work capacity, age and health status, and may influence workers’ attitude toward taking SA.

## Conclusion

The results of this study support associations previously observed between long-term back-pain SA and later DP. We further identified a modest association between back-pain SA and EAP. Exposure to heavy physical workload or low job control was associated with DP showing a dose–response trend, whereas exposure to higher job demands was associated with a decreased risk. The small and inconsistent associations of physical workload, job control and demands with EAP across genders suggest that they play a minor role in exiting from the labour force through this route, if any.

The joint effect of back-pain SA and work factors on DP risk appears mainly additive, with only a negative effect modification found for job strain in women. However, the decrease in the risk gradient by increasing exposure to physical workload and low job control among men with more back-pain SA episodes or days suggests that when back pain becomes more persistent and disabling its effect on the risk of DP would be less dependent on working conditions.

## Supplementary Information

Below is the link to the electronic supplementary material.

Supplementary file1 (DOCX 21 KB)

## Data Availability

The data that support the findings of this study are available from Karolinska Institutet but restrictions apply to the availability of these data, which were used under license for the current study, and so are not publicly available. Aggregated data are however available from the authors upon request and with permission of Karolinska Institutet.

## References

[CR1] Almroth M, Hemmingsson T, Sörberg Wallin A, Kjellberg K, Burström B, Falkstedt D (2021). Psychosocial working conditions and the risk of diagnosed depression: a Swedish register-based study. Psychol Med.

[CR2] Badarin K, Hemmingsson T, Hillert L, Kjellberg K (2021). Physical workload and increased frequency of musculoskeletal pain: a cohort study of employed men and women with baseline occasional pain. Occup Environ Med.

[CR3] Borg K, Hensing G, Alexanderson K (2004). Risk factors for disability pension over 11 years in a cohort of young persons initially sick-listed with low back, neck, or shoulder diagnoses. Scand J Public Health.

[CR4] Bot SD, van der Waal JM, Terwee CB, van der Windt DA, Schellevis FG, Bouter LM, Dekker J (2005). Incidence and prevalence of complaints of the neck and upper extremity in general practice. Ann Rheum Dis.

[CR5] Browne P, Carr E, Fleischmann M, Xue B, Stansfeld SA (2018). The relationship between workplace psychosocial environment and retirement intentions and actual retirement: a systematic review. Eur J Ageing.

[CR6] Canivet C, Choi B, Karasek R, Moghaddassi M, Staland-Nyman C, Östergren PO (2013). Can high psychological job demands, low decision latitude, and high job strain predict disability pensions? A 12-year follow-up of middle-aged Swedish workers. Int Arch Occup Environ Health.

[CR7] Carstensen J, Andersson D, André M, Engström S, Magnusson H, Borgquist LA (2012). How does comorbidity influence healthcare costs? A population-based cross-sectional study of depression, back pain and osteoarthritis. BMJ Open.

[CR8] Choi BK, Clays E, De Bacquer D, Karasek R (2008). Socioeconomic status, job strain and common mental disorders—an ecological (occupational) approach. Scand J Work Environ Health Suppl.

[CR9] da Costa BR, Vieira ER (2010). Risk factors for work-related musculoskeletal disorders: a systematic review of recent longitudinal studies. Am J Ind Med.

[CR10] d'Errico A, Cardano M, Landriscina T, Marinacci C, Pasian S, Petrelli A, Costa G (2011). Workplace stress and prescription of antidepressant medications: a prospective study on a sample of Italian workers. Int Arch Occup Environ Health.

[CR11] Dorner TE, Alexanderson K, Svedberg P, Ropponen A, Stein KV, Mittendorfer-Rutz E (2015). Sickness absence due to back pain or depressive episode and the risk of all-cause and diagnosis-specific disability pension: a Swedish cohort study of 4,823,069 individuals. Eur J Pain.

[CR12] Driscoll T, Jacklyn G, Orchard J, Passmore E, Vos T, Freedman G, Lim S, Punnett L (2014). The global burden of occupationally related low back pain: estimates from the Global Burden of Disease 2010 study. Ann Rheum Dis.

[CR13] Falkstedt D, Hemmingsson T, Albin M, Bodin T, Ahlbom A, Selander J, Gustavsson P, Andersson T, Almroth M, Kjellberg K (2021). Disability pensions related to heavy physical workload: a cohort study of middle-aged and older workers in Sweden. Int Arch Occup Environ Health.

[CR14] Forsakringskassan.se (2020) Sickness compensation. The Swedish Social Insurance Agency. https://www.forsakringskassan.se/. Accessed 5 Nov 2021

[CR15] Friis K, Ekholm O, Hundrup YA, Obel EB, Grønbaek M (2007). Influence of health, lifestyle, working conditions, and sociodemography on early retirement among nurses: the Danish Nurse Cohort Study. Scand J Public Health.

[CR16] Friis K, Ekholm O, Hundrup YA (2008). The relationship between lifestyle, working environment, socio-demographic factors and expulsion from the labour market due to disability pension among nurses. Scand J Caring Sci.

[CR17] Gourmelen J, Chastang JF, Ozguler A, Lanoë JL, Ravaud JF, Leclerc A (2007). Frequency of low back pain among men and women aged 30 to 64 years in France. Results of two national surveys. Ann Readapt Med Phys.

[CR18] Gouveia N, Rodrigues A, Eusébio M, Ramiro S, Machado P, Canhão H, Branco JC (2016). Prevalence and social burden of active chronic low back pain in the adult Portuguese population: results from a national survey. Rheumatol Int.

[CR19] Hasselhorn HM, Apt W (2015). Understanding employment participation of older workers: creating a knowledge base for future labour market challenges.

[CR20] Jensen LD, Ryom PK, Christensen MV, Andersen JH (2012). Differences in risk factors for voluntary early retirement and disability pension: a 15-year follow-up in a cohort of nurses' aides. BMJ Open.

[CR21] Kadefors R, Nilsson K, Östergren PO, Rylander L, Albin M (2019). Social inequality in working life expectancy in Sweden. Z Gerontol Geriatr.

[CR22] Kausto J, Miranda H, Pehkonen I, Heliövaara M, Viikari-Juntura E, Solovieva S (2011). The distribution and co-occurrence of physical and psychosocial risk factors for musculoskeletal disorders in a general working population. Int Arch Occup Environ Health.

[CR23] Khan AN, Jacobsen HE, Khan J, Filippi CG, Levine M, Lehman RA, Riew KD, Lenke LG, Chahine NO (2017). Inflammatory biomarkers of low back pain and disc degeneration: a review. Ann N Y Acad Sci.

[CR24] Kjellberg K, Lundin A, Falkstedt D, Allebeck P, Hemmingsson T (2016). Long-term physical workload in middle age and disability pension in men and women: a follow-up study of Swedish cohorts. Int Arch Occup Environ Health.

[CR25] Knardahl S, Johannessen HA, Sterud T, Härmä M, Rugulies R, Seitsamo J, Borg V (2017). The contribution from psychological, social, and organizational work factors to risk of disability retirement: a systematic review with meta-analyses. BMC Public Health.

[CR26] Knezevic NN, Candido KD, Vlaeyen JWS, Van Zundert J, Cohen SP (2021). Low back pain. Lancet.

[CR27] Krause N, Lynch J, Kaplan GA, Cohen RD, Goldberg DE, Salonen JT (1997). Predictors of disability retirement. Scand J Work Environ Health.

[CR28] Krokstad S, Johnsen R, Westin S (2002). Social determinants of disability pension: a 10-year follow-up of 62 000 people in a Norwegian county population. Int J Epidemiol.

[CR29] Lahelma E, Laaksonen M, Lallukka T, Martikainen P, Pietiläinen O, Saastamoinen P, Gould R, Rahkonen O (2012). Working conditions as risk factors for disability retirement: a longitudinal register linkage study. BMC Public Health.

[CR30] Laires PA, Canhão H, Rodrigues AM, Eusébio M, Gouveia M, Branco JC (2018). The impact of osteoarthritis on early exit from work: results from a population-based study. BMC Public Health.

[CR31] Lallukka T, Mänty M, Cooper C, Fleischmann M, Kouvonen A, Walker-Bone KE, Head JA, Halonen JI (2018). Recurrent back pain during working life and exit from paid employment: a 28-year follow-up of the Whitehall II Study. Occup Environ Med.

[CR32] Laun L, Palme M, Coile CC, Milligan K, Wise DA (2019). The recent rise of labor force participation of older workers in Sweden. Social security programs and retirement around the world: working longer.

[CR33] Lund T, Villadsen E (2005). Who retires early and why? Determinants of early retirement pension among Danish employees 57–62 years. Eur J Ageing.

[CR34] Lund T, Iversen L, Poulsen KB (2001). Work environment factors, health, lifestyle and marital status as predictors of job change and early retirement in physically heavy occupations. Am J Ind Med.

[CR35] MacDonald LA, Karasek RA, Punnett L, Scharf T (2001). Covariation between workplace physical and psychosocial stressors: evidence and implications for occupational health research and prevention. Ergonomics.

[CR36] Maher C, Underwood M, Buchbinder R (2017). Non-specific low back pain. Lancet.

[CR37] Mäki NE, Martikainen PT, Eikemo T, Menvielle G, Lundberg O, Ostergren O, Mackenbach JP (2014). EURO-GBD-SE consortium members. The potential for reducing differences in life expectancy between educational groups in five European countries: the effects of obesity, physical inactivity and smoking. J Epidemiol Community Health.

[CR38] Mather L, Ropponen A, Mittendorfer-Rutz E, Narusyte J, Svedberg P (2019). Health, work and demographic factors associated with a lower risk of work disability and unemployment in employees with lower back, neck and shoulder pain. BMC Musculoskelet Disord.

[CR39] Nieminen LK, Pyysalo LM, Kankaanpää MJ (2021). Prognostic factors for pain chronicity in low back pain: a systematic review. Pain Rep.

[CR40] Nygaard PP, Skovlund SV, Sundstrup E, Andersen LL (2020). Is low-back pain a limiting factor for senior workers with high physical work demands? A cross-sectional study. BMC Musculoskelet Disord.

[CR41] Oberlinner C, Yong M, Nasterlack M, Pluto RP, Lang S (2015). Combined effect of back pain and stress on work ability. Occup Med (Lond).

[CR42] OECD (2019) OECD Stat. Short-term labour market statistics. https://stats.oecd.org/Index.aspx?DataSetCode=STLABOUR. Accessed 08 Nov 2021

[CR43] Øverås CK, Johansson MS, de Campos TF, Ferreira ML, Natvig B, Mork PJ, Hartvigsen J (2021). Distribution and prevalence of musculoskeletal pain co-occurring with persistent low back pain: a systematic review. BMC Musculoskelet Disord.

[CR44] Palmer KT, Goodson N (2015). Ageing, musculoskeletal health and work. Best Pract Res Clin Rheumatol.

[CR45] Parsons S, Breen A, Foster NE, Letley L, Pincus T, Vogel S, Underwood M (2007). Prevalence and comparative troublesomeness by age of musculoskeletal pain in different body locations. Fam Pract.

[CR46] Prakash KC, Neupane S, Leino-Arjas P, von Bonsdorff MB, Rantanen T, von Bonsdorff ME, Seitsamo J, Ilmarinen J, Nygård CH (2017). Work-related biomechanical exposure and job strain in midlife separately and jointly predict disability after 28 years: a Finnish longitudinal study. Scand J Work Environ Health.

[CR47] Punnett L, Wegman DH (2004). Work-related musculoskeletal disorders: the epidemiologic evidence and the debate. J Electromyogr Kinesiol.

[CR48] Rahman S, Mittendorfer-Rutz E, Dorner TE, Pazarlis K, Ropponen A, Svedberg P, Wang M, Helgesson M (2019). Work-disability in low back pain patients with or without surgery, and the role of social insurance regulation changes in Sweden. Eur J Public Health.

[CR49] Reeuwijk KG, van Klaveren D, van Rijn RM, Burdorf A, Robroek SJ (2017). The influence of poor health on competing exit routes from paid employment among older workers in 11 European countries. Scand J Work Environ Health.

[CR50] Rice NE, Lang IA, Henley W, Melzer D (2011). Common health predictors of early retirement: findings from the English Longitudinal Study of Ageing. Age Ageing.

[CR51] Risbud MV, Shapiro IM (2014). Role of cytokines in intervertebral disc degeneration: pain and disc content. Nat Rev Rheumatol.

[CR52] Robroek SJ, Schuring M, Croezen S, Stattin M, Burdorf A (2013). Poor health, unhealthy behaviors, and unfavorable work characteristics influence pathways of exit from paid employment among older workers in Europe: a four year follow-up study. Scand J Work Environ Health.

[CR53] scb.se (2021) Price Base Amount. Statistiska centralbyrån [Statistics Sweden]. https://www.scb.se/en. Accessed 08 Nov 2021

[CR54] Schuring M, Robroek SJ, Otten FW, Arts CH, Burdorf A (2013). The effect of ill health and socioeconomic status on labor force exit and re-employment: a prospective study with ten years follow-up in the Netherlands. Scand J Work Environ Health.

[CR55] Singh V, Manchikanti L, Onyewu O, Benyamin RM, Datta S, Geffert S, Parr AT, Falco FJ (2012). An update of the appraisal of the accuracy of thoracic discography as a diagnostic test for chronic spinal pain. Pain Physician.

[CR56] Sundstrup E, Andersen LL (2017). Hard physical work intensifies the occupational consequence of physician-diagnosed back disorder: prospective cohort study with register follow-up among 10,000 workers. Int J Rheumatol.

[CR57] Sundstrup E, Hansen ÅM, Mortensen EL, Poulsen OM, Clausen T, Rugulies R, Møller A, Andersen LL (2018). Retrospectively assessed physical work environment during working life and risk of sickness absence and labour market exit among older workers. Occup Environ Med.

[CR58] Thielen K, Nygaard E, Andersen I, Diderichsen F (2014). Employment consequences of depressive symptoms and work demands individually and combined. Eur J Public Health.

[CR59] Thomas E, Peat G, Croft P (2014). Defining and mapping the person with osteoarthritis for population studies and public health. Rheumatology (Oxford).

[CR60] Thorsen SV, Jensen PH, Bjørner JB (2016). Psychosocial work environment and retirement age: a prospective study of 1876 senior employees. Int Arch Occup Environ Health.

[CR61] Van Boxem K, Cheng J, Patijn J, van Kleef M, Lataster A, Mekhail N, Van Zundert J (2010). 11. Lumbosacral radicular pain. Pain Pract.

[CR62] van den Berg TI, Elders LA, Burdorf A (2010). Influence of health and work on early retirement. J Occup Environ Med.

[CR63] van Rijn RM, Robroek SJ, Brouwer S, Burdorf A (2014). Influence of poor health on exit from paid employment: a systematic review. Occup Environ Med.

[CR64] Vatcheva KP, Lee M, McCormick JB, Rahbar MH (2016). Multicollinearity in regression analyses conducted in epidemiologic studies. Epidemiology (Sunnyvale).

[CR65] Venti S, Wise DA (2015). The long reach of education: early retirement. J Econ Ageing.

[CR66] Yu S, Lu ML, Gu G, Zhou W, He L, Wang S (2015). Association between psychosocial job characteristics and sickness absence due to low back symptoms using combined DCS and ERI models. Work.

