# Supplementary material for: Long-term sick leave for back pain, exposure to physical workload and psychosocial factors at work, and risk of disability and early-age retirement among aged Swedish workers
Source: Int Arch Occup Environ Health. 2022 Apr 22;95(7):1521–35. doi: 10.1007/s00420-022-01862-8 (PMC9424129; doi:10.1007/s00420-022-01862-8)
Supplement: Supplementary file 1 — Supplementary file1 (DOCX 21 KB) [file 420_2022_1862_MOESM1_ESM.docx]

**Supplementary Tables**

**Table S1** Hazard ratios and 95% confidence intervals for disability pension according to back pain sickness days

|  |  | **N cases (%)** | **Model 1** | **Model 2** |
| --- | --- | --- | --- | --- |
| **Men** |  |  |  |  |
| Back-pain SA | None | 10,198 (2) | 1 | 1 |
|  | <90 days | 407 (4) | 1.80 (1.63-1.98) | 1.55 (1.41-1.72) |
|  | >90 days | 567 (14) | 6.10 (5.60-6.63) | 5.05 (4.64-5.50) |
| **Women** |  |  |  |  |
| Back-pain SA | None | 13,019 (3) | 1 | 1 |
|  | <90 days | 491 (5) | 1.63 (1.49-1.79) | 1.51 (1.38-1.66) |
|  | >90 days | 656 (14) | 4.45 (4.11-4.81) | 4.06 (3.75-4.39) |

*Back-pain SA = n. days of sickness absence for back pain*

*Model 1 is adjusted for age*

*Model 2 is adjusted for age, civil status, previous unemployment, and highest achieved education.*

**Table S2** Hazard ratios and 95% confidence intervals for early old age pension without income (1 PBA) according to back pain sickness days

|  |  | **N cases (%)** | **Model 1** | **Model 2** |
| --- | --- | --- | --- | --- |
| **Men** |  |  |  |  |
| Back-pain SA | None | 28,879 (18) | 1 | 1 |
|  | <90 days | 753 (21) | 1.20 (1.11-1.29) | 1.11 (1.03-1.19) |
|  | >90 days | 340 (20) | 1.18 (1.06-1.32) | 1.07 (0.96-1.19) |
| **Women** |  |  |  |  |
| Back-pain SA | None | 28,466 (18) | 1 | 1 |
|  | <90 days | 777 (22) | 1.23 (1.14-1.32) | 1.17 (1.09-1.25) |
|  | >90 days | 408 (22) | 1.19 (1.16-1.22) | 1.15 (1.05-1.27) |

*Back-pain SA = n. days of sickness absence for back pain*

*Model 1 is adjusted for age*

*Model 2 is adjusted for age, civil status, previous unemployment, and highest achieved education.*

**Table S3** Hazard ratios and 95% confidence intervals for disability pension according to *combinations of* low back pain sickness days and physical and psychosocial workplace factors

| **Back-pain SA** |  | **None** | **<90 days** | **>90 days** | **P-value** |
| --- | --- | --- | --- | --- | --- |
| **Men** |  |  |  |  |  |
| Physical workload | Low | 1 | 1.59 (1.13-2.22) | 5.25 (3.87-7.13) |  |
|  | Med | 1.41 (1.33-1.49) | 2.05 (1.70-2.47) | 7.52 (6.48-8.73) |  |
|  | High | 1.69 (1.59-1.79) | 2.48 (2.17-2.84) | 7.59 (6.73-8.56) | 0.4097 |
| Job strain | Low | 1 | 1.45 (1.23-1.71) | 4.70 (4.09-5.41) |  |
|  | Med | 0.93 (0.88-0.97) | 1.32 (1.09-1.61) | 4.68 (3.98-5.50) |  |
|  | High | 1.01 (0.97-1.06) | 1.56 (1.32-1.83) | 4.82 (4.18-5.54) | 0.8649 |
| Job control | Low | 1.56 (1.48.1.64) | 2.23 (1.93-2.58) | 7.30 (6.44-8.29) |  |
|  | Med | 1.43 (1.35-1.51) | 2.17 (1.85-2.56) | 6.69 (5.80-7.72) |  |
|  | High | 1 | 1.67 (1.26-2.22) | 6.37 (5.19-9.96) | 0.1288 |
| Job demands | Low | 1 | 1.38 (1.21-1.58) | 4.74 (4.24-5.31) |  |
|  | Med | 0.75 (0.72-0.79) | 1.31 (1.09-1.57) | 3.70 (3.13-4.38) |  |
|  | High | 0.71 (0.67-0.75) | 1.12 (0.87-1.44) | 3.97 (3.27-4.82) | 0.2052 |
| **Women** |  |  |  |  |  |
| Physical workload | Low | 1 | 1.59 (1.22-2.06) | 3.97 (3.18-4.95) |  |
|  | Med | 1.32 (1.26-1.39) | 1.77 (1.48-2.11) | 5.42 (4.70-6.26) |  |
|  | High | 1.70 (1.62-1.78) | 2.40 (2.13-2.71) | 6.20 (5.56-6.92) | 0.5579 |
| Job strain | Low | 1 | 1.82 (1.55-2.13) | 4.14 (3.57-4.80) |  |
|  | Med | 1.04 (0.99-1.08) | 1.34 (1.14-1.57) | 4.03 (3.52-4.61) |  |
|  | High | 1.04 (0.99-1.09) | 1.31 (1-13-1.53) | 3.66 (3.21-4.18) | 0.0010 |
| Job control | Low | 1.55 (1.48-1.62) | 2.11 (1.85-2.41) | 5.65 (5.02-6.35) |  |
|  | Med | 1.47 (1.41-1.54) | 2.09 (1.80-2.43) | 6.03 (5.31-6.85) |  |
|  | High | 1 | 1.78 (1.41-2.24) | 4.09 (3.33-5.03) | 0.2122 |
| Job demands | Low | 1 | 1.50 (1.32-1.71) | 3.75 (3.33-4.22) |  |
|  | Med | 0.81 (0.77-0.84) | 1.22 (1.05-1.41) | 3.67 (3.24-4.16) |  |
|  | High | 0.75 (0.71-0.79) | 1.10 (0.88-1.38) | 2.78 (2.29-3.37) | 0.2214 |

*Back-pain SA = n. days of sickness absence for back pain*

*Models adjusted for age, civil status, previous unemployment, and education*

*P-value corresponds to Wald test for interaction term*

**Table S4** Hazard ratios and 95% confidence intervals for early old age pension without income (1 PBA) according to *combinations of* low back pain sickness days and physical and psychosocial workplace factors

| **SA-BP** |  | **None** | **<90 days** | **>90 days** | **P-Value** |
| --- | --- | --- | --- | --- | --- |
| **Men** |  |  |  |  |  |
| Physical workload | Low | 1 | 1.15 (0.96-1.38) | 1.45 (1.13-1.88) |  |
|  | Med | 0.93 (0.91-0.96) | 1.01 (0.89-1.14) | 1.14 (0.95-1.36) |  |
|  | High | 0.82 (0.79-0.85) | 0.96 (0.87-1.07) | 0.78 (0.66-0.91) | 0.0353 |
| Job strain | Low | 1 | 1.11 (0.98-1.25) | 0.97 0.81-1.17) |  |
|  | Med | 1.13 (1.10-1.17) | 1.22 (1.07-1.38) | 1.35 (1.11-1.63) |  |
|  | High | 1.02 (0.99-1.05) | 1.18 (1.04-1.34) | 1.14 (0.95-1.37) | 0.5265 |
| Job control | Low | 0.83 (0.80-0.85) | 1.00 (0.90-1.12) | 0.93 (0.78-1.10) |  |
|  | Med | 0.91 (0.89-0.94) | 0.96 (0.85-1.08) | 0.96 (0.81-1.13) |  |
|  | High | 1 | 1.13 (0.96-1.33) | 1.12 (0.88-1.44) | 0.4908 |
| Job demands | Low | 1 | 1.18 (1.06-1.30) | 1.01 (0.86-1.18) |  |
|  | Med | 1.05 (1.02-1.08) | 1.15 (1.01-1.32) | 1.25 (1.04-1.51) |  |
|  | High | 1.14 (1.11-1.18) | 1.20 (1.01-1.41) | 1.27 (1.01-1.60) | 0.4625 |
| **Women** |  |  |  |  |  |
| Physical workload | Low | 1 | 1.19 (1.00-1.07) | 1.24 (0.99-1.56) |  |
|  | Med | 1.09 (1.06-1.13) | 1.20 (1.05-1.37) | 1.15 (0.96-1.37) |  |
|  | High | 1.04 (1.00-1.07) | 1.23 (1.11-1.36) | 1.23 (1.07-1.41) | 0.6101 |
| Job strain | Low | 1 | 1.14 (0.99-1.31) | 1.16 (0.97-1.40) |  |
|  | Med | 1.03 (1.00-1.06) | 1.25 (1.11-1.40) | 1.15 (0.97-1.36) |  |
|  | High | 1.06 (1.03-1.10) | 1.19 (1.06-1.34) | 1.24 (1.06-1.45) | 0.8851 |
| Job control | Low | 1.08 (1.05-1.12) | 1.26 (1.13-1.39) | 1.29 (1.12-1.48) |  |
|  | Med | 1.06 (1.03-1.10) | 1.23 (1.13-1.39) | 1.10 (0.93-1.31) |  |
|  | High | 1 | 1.13 (0.95-1.34) | 1.24 (0.99-1.55) | 0.7111 |
| Job demands | Low | 1 | 1.23 (1.10-1.36) | 1.10 (0.95-1.29) |  |
|  | Med | 0.94 (0.91-0.97) | 1.06 (0.94-1.19) | 1.13 (0.97-1.32) |  |
|  | High | 0.97 (0.93-1.00) | 1.07 (0.90-1.26) | 1.19 (1.16-1.22) | 0.7009 |

*PBA=price basic amount; Back-pain SA = n. days of sickness absence for back pain*

*Models adjusted for age, civil status, previous unemployment, and education*

*P-value corresponds to Wald test for interaction term*
